# Effects of chokeberry pomace supplementation on biochemical, metabolic and antioxidant parameters in high-yielding dairy goats

**DOI:** 10.2478/jvetres-2025-0054

**Published:** 2025-10-04

**Authors:** Monika Szymańska-Czerwińska, Krzysztof Niemczuk, Nina Strzałkowska, Zbigniew Osiński, Agnieszka Wierzbicka, Barbara Wijas, Sylwester Marczak, Karina Horbańczuk, Artur Jóźwik

**Affiliations:** 1 Department of Biotechnology and Nutrigenomics Poland; 2 Department of Virology and Viral Animal Diseases, National Veterinary Research Institute, 24-100 Puławy, Poland; 3 Department of Technique and Food Development, Faculty of Human Nutrition and Consumer Sciences, Warsaw University of Life Sciences (WULS-SGGW), 02-776 Warsaw, Poland; 4 Institute of Genetics and Animal Biotechnology, Polish Academy of Sciences, 05-552 Magdalenka, Poland

**Keywords:** bioactives, biochemical parameters, feed additives, goat farming, oxidative stress

## Abstract

**Introduction:**

The aim of the study was to evaluate the effects of dietary supplementation with dried black chokeberry pomace (*Aronia melanocarpa*) on metabolic and antioxidant parameters in high-yielding dairy goats.

**Material and Methods:**

Twenty-seven Polish White Improved goats in mid-lactation were divided into three groups: a control group and two experimental groups receiving chokeberry pomace at 15 g/kg (A1) and 30 g/kg (A2) of dry matter in the feed. Biochemical, enzymatic, mineral, and oxidative stress parameters were analysed.

**Results:**

Supplementation, particularly in group A2, improved the lipid profile by reducing low-density lipoprotein and triglycerides and increasing high-density lipoprotein levels. It also enhanced antioxidant capacity (higher glutathione and 2,2-diphenyl-1-picrylhydrazyl values) and positively influenced liver and lysosomal enzyme activity. Changes in creatinine, cholinesterase and creatine kinase levels were observed, along with correlations between calcium and metabolic markers.

**Conclusion:**

These findings suggest that chokeberry pomace may serve as a valuable feed additive in the nutrition of high-yielding dairy goats, promoting their metabolic health and potentially enhancing milk quality.

## Introduction

In recent years, there has been growing interest in the use of natural feed additives with health-promoting properties in the nutrition of ruminants. A prominent example is black chokeberry (*Aronia melanocarpa*), a plant with exceptionally high antioxidant potential, rich in polyphenols, anthocyanins, flavonoids and vitamins (10). Poland is one of the leading global producers of chokeberry, and has exploitable processing by-products, such as dried pomace, which may serve as a valuable source of bioactive compounds in animal feeding.

Previous studies conducted in both animal models and human populations have demonstrated that chokeberry may have beneficial effects on liver function, lipid metabolism, metabolic parameters and redox-inflammatory homeostasis (5, 14, 21, 24). Because of their intensive milk production and high metabolic activity, high-yielding dairy goats are particularly susceptible to oxidative stress (21) and dysfunction of parenchymal organs and biochemical imbalances (17). The inclusion of natural plant-based supplements in their diet may support physiological functions, improve animal welfare and enhance milk quality. Despite the health-promoting properties of chokeberry being well documented, its effects on the physiology of high-yielding dairy goats have not yet been shown in exhaustive scientific data. High-producing dairy goats represent a rarely used, yet valuable model for evaluating the effects of antioxidant supplementation because of their intensive metabolic turnover and the physiological demands on them during lactation.

The aim of this study was to assess the effect of dietary supplementation with dried chokeberry pomace at two different doses on selected blood biochemical parameters in high-yielding dairy goats. The analysis included indicators of liver, kidney and muscle function; lipid profile; carbohydrate and energy metabolism (glucose and lactate); concentrations of selected macro- and microelements; enzymatic activity (aminopeptidases and lysosomal enzymes); and oxidative stress markers. Correlation analysis among these parameters was also performed to identify potential mechanisms of action of chokeberry bioactive compounds.

## Material and Methods

### Livestock and diet

The feeding trial design, animal housing and the source of chokeberry pomace were described in detail previously (21). Briefly, 27 dairy goats (Polish White Improved breed) in mid-lactation were randomly assigned to three dietary groups (n = 9): a control group (standard diet) and two experimental groups receiving 15 g/kg (group A1) or 30 g/kg (group A2) of dried chokeberry pomace, based on the dry matter content of the feed.

Blood samples were collected from the jugular vein by licensed veterinarians, following standard veterinary procedures and Polish animal welfare regulations (Act of 15 January 2015 on the Protection of Animals Used for Scientific or Educational Purposes, Journal of Laws 2015, item 266). According to this legislation, the procedures used in this study did not require the approval of the Local Ethics Committee for Animal Experimentation.

A volume of 9 mL of blood was drawn using a disposable vacuum system with a 20 G needle into tubes without anticoagulant. Samples were stored at 4°C for 30–45 min to allow clotting and were then centrifuged at 3,000 rpm for 10 min to obtain serum. Serum was analysed immediately or stored according to the manufacturer’s instructions. Samples intended for lysosomal enzyme analysis were stored at –20°C until use.

For serum collection, blood was drawn into 1.8 mL microtubes containing 3.2% buffered sodium citrate (Becton Dickinson, Franklin Lakes, NJ, USA) and centrifuged at 4,750 × *g* for 10 min. Serum samples were immediately frozen at –70°C. All biochemical analyses were performed using the semi-automated Cobas Integra 400 plus analyser (Roche Diagnostics, Rotkreuz, Switzerland). Reagent codes were entered according to the manufacturer’s instructions. The following biochemical parameters were assessed: albumin (ALB), alkaline phosphatase (ALP), alanine aminotransferase (ALT), aspartate aminotransferase (AST), total bilirubin (BILT), calcium, cholinesterase (CHE), total cholesterol (CHOL), creatine kinase (CK), chloride, creatinine (CRE), iron (Fe), gamma-glutamyltransferase (GGT), glucose (GLU), high-density lipoprotein (HDL), potassium, lactate (LACT) lactate dehydrogenase (LDH), low-density lipoprotein (LDL), lysosomal acid lipase (LIP), magnesium, sodium, phosphorus, total protein (TP) and triglycerides (TRIG).

### Enzyme activity

Lysosomal and aminopeptidase activities were determined using substrates from Sigma-Aldrich (St. Louis, MO, USA). Activities of β-galactosidase (β-GAL; EC 3.2.1.23), α-glucosidase (α-GLU; EC 3.2.1.22), β-glucosidase (β-GLU; EC 3.2.1.21), β-glucuronidase (β-GRD; EC 3.2.1.31), N-acetyl-β-hexosaminidase (HEX; EC 3.2.1.52) and mannosidase (MAN; EC 3.2.1.24) were assayed according to Barrett and Heath (2), using p-nitrophenyl substrates and incubation at 37°C. Acid phosphatase (ACP; EC 3.1.3.2) activity was measured using 4-nitrophenyl phosphate with absorbance read at 420 nm. Activities of alanyl-aminopeptidase (AlaAP), leucyl-aminopeptidase (LeuAP) and arginyl -aminopeptidase (ArgAP) were determined in supernatants according to McDonald and Barrett (13), using Fast Blue BB salt derivatives, with absorbance measured at 540 nm.

### Vitamin C

Vitamin C content was determined according to Omaye *et al*. (15), using a LambdaBio-20 spectrophotometer (Perkin Elmer, Shelton, CT, USA).

### Glutathione assay

Reduced glutathione (GSH) was measured using the OxisResearch Bioxytech GSH/GSSG (glutathione disulphide)-412 assay (Oxis International, Foster City, CA, USA). Samples were frozen with 1-methyl-2-vinylpyridinium at –80°C, thawed to induce erythrolysis, and processed following the manufacturer’s protocol using a Synergy4 microplate reader and Gen5 software (both Agilent, Winooski, VT, USA). Results were expressed in μM.

### 2,2-diphenyl-1-picrylhydrazyl (DPPH) radical scavenging capacity

The DPPH free-radical scavenging activity of antioxidants in the serum samples was measured using a modified method by Brand-Williams *et al*. (3). Serum (0.2 mL) was mixed with 2 mL of methanol containing 1% acetic acid, homogenised, incubated at 40°C for 2 h and centrifuged. The supernatant (0.5 mL) was reacted with ethanolic DPPH solution, and absorbance at 517 nm was measured using a Cary 50 Bio UV-VIS spectrophotometer (Agilent, Mulgrave, VIC, Australia).

### Total phenolic content

Total phenolic content (POL) was determined using a modified Folin–Ciocalteu method (20). After incubation at 40°C, absorbance was measured at 765 nm. Results were calculated based on a gallic acid equivalent (GAE) standard curve and expressed as mg GAE/mL.

### Statistical analysis

Data were presented as mean ± standard deviation (SD). Normality of distribution was assessed using the Shapiro–Wilk test in Statistica 13 (TIBCO, Palo Alto, CA, USA). For data showing a normal distribution, statistical significance was evaluated by two-factor fixed model analysis of variance (ANOVA), followed by the least significant difference *post-hoc* test. Differences with P-value ≤ 0.05 were considered statistically significant.

To assess the effect of chokeberry supplementation on biochemical parameters, ANOVA included the control group to determine between-group differences. Additionally, Pearson’s correlation analysis was conducted within the supplemented groups (A1 and A2) to assess relationships between parameter values at the beginning and end of the experiment. Baseline values referred to pre-supplementation measurements within each group. The control group was excluded from correlation analysis, as its inclusion could distort the correlation structure: animals in this group did not receive supplementation, and observed changes could reflect natural physiological dynamics rather than effects of the additive. This approach enabled a comprehensive evaluation of both between-group effects (*via* ANOVA) and within-group dynamics (*via* correlation analysis).

## Results

### Metabolic and renal function parameters

Table 1 presents the results of metabolic and renal function parameters as changes over the course of the experiment. Serum ALB concentrations decreased in all groups, with statistically significant reductions observed in group A1 (P-value = 0.044) and the control group (P-value = 0.006), but not in group A2. All groups experienced significant GGT activity falls (P-value < 0.05). In group A1, a significant decrease in ALP activity was noted (P-value = 0.046), whereas in group A2, there was an increase but not a significant one. The only statistically significant decline in AST activity was in group A1 (P-value = 0.006), while the falls in ALT activity were not significant in any group. The BILT concentration remained unchanged. A significant decrease in LIP activity was observed in group A1 (P-value = 0.010) and group A2 (P-value = 0.005). It was in group A1 again and in the control group where CHE activity was significantly lower at the experiment’s end (P-value = 0.001 and P-value = 0.014, respectively). The TP levels decreased in all groups, but without statistical significance. The measured CK activity rose significantly in group A2 (P-value < 0.001), fell in the control group (P-value = 0.032) and remained unchanged in group A1. The concentrations of CRE and GLU showed no significant changes. Observation of significantly lower LDH activity was made at the final measurement in group A2 (P-value < 0.001). The levels of LACT significantly decreased in all groups (P-value < 0.001).

**Table 1.** Metabolic and renal function parameters in blood serum of lactating goats after supplementation with *Aronia melanocarpa* pomace

Parameter	Pomace mass per kg feed (group)	Mean ± standard deviation		P-value
		Start	Finish	A1, A2, C Start to finish
ALB g/L	15g (A1)	41.7±3.82	38.5±2.51	0.044*
	30g (A2)	40.1±1.87	39.0±1.29	0.483
	control (C)	42.2±5.6	37.6±3.36	0.006*
GGT IU/L	15g (A1)	51.5±5.68	45.4±4.78	0.012*
	30g (A2)	51.7±3.40	41.3±4.12	<0.001*
	control (C)	59.2±5.3	54.2±6.21	0.042*
ALP IU/L	15g (A1)	124.7±43.69	99.4±11.96	0.046*
	30g (A2)	110.1±26.08	115.7±23.78	0.645
	control (C)	119.2±27.0	102.0±6.95	0.184
ALT IU/L	15g (A1)	17.2±2.05	16.3±1.94	0.364
	30g (A2)	16.4±1.59	14.9±2.08	0.109
	control (C)	18.6±2.4	18.0±2.20	0.581
AST IU/L	15g (A1)	89.2±8.63	79.7±8.31	0.006*
	30g (A2)	78.1±4.83	74.8±5.69	0.312
	control (C)	91.3±7.2	90.1±7.06	0.726
BILT mg/dl	15g (A1)	0.8±0.20	0.9±0.14	0.310
	30g (A2)	0.7±0.18	0.6±0.19	0.127
	control (C)	0.9±0.2	0.9±0.19	0.975
LIP IU/L	15g (A1)	19.8±2.23	17.4±1.85	0.010*
	30g (A2)	19.1±1.90	16.5±1.81	0.005*
	control (C)	19.8±2.1	19.0±1.57	0.366
CHE IU/L	15g (A1)	73.9±14.51	53.8±8.89	0.001*
	30g (A2)	52.8±13.74	57.0±13.25	0.453
	control (C)	70.2±11.7	55.1±9.14	0.014*
TP g/L	15g (A1)	72.7±5.26	70.2±5.62	0.345
	30g (A2)	71.1±5.41	69.8±4.13	0.610
	control (C)	75.4±6.4	72.3±6.30	0.249
CK IU/L	15g (A1)	183.8±20.81	158.8±20.84	0.077
	30g (A2)	146.6±17.56	214.7±50.78	<0.001*
	control (C)	181.4±29.0	150.0±16.71	0.032*
LDH IU/L	15g (A1)	801.3±50.46	778.2±37.45	0.458
	30g (A2)	779.6±53.64	638.9±48.52	<0.001*
	control (C)	798.9±106.6	810.6±75.38	0.714
CRE μmol/L	15g (A1)	60.0±6.26	59.3±3.02	0.761
	30g (A2)	64.2±3.70	61.7±6.09	0.255
	control (C)	61.7±3.6	57.3±4.24	0.062
GLU mmol/L	15g (A1)	3.1±0.41	3.1±0.18	0.609
	30g (A2)	3.2±0.22	3.4±0.31	0.266
	control (C)	3.1±0.4	3.3±0.24	0.289
LACT mmol/L	15g (A1)	2.3±0.17	1.5±0.21	<0.001*
	30g (A2)	2.6±0.47	1.6±0.31	<0.001*
	control (C)	2.7±0.6	1.7±0.38	<0.001*

* – statistically significant differences at P-value ≤ 0.05

1 ALB – albumin; GGT– gamma-glutamyltransferase; ALP – alkaline phosphatase; ALT – alanine aminotransferase; AST – aspartate aminotransferase; BILT – total bilirubin; LIP – lipase; CHE – cholinesterase; TP – total protein; CK – creatine kinase; LDH – lactate dehydrogenase; CRE – creatinine; GLU – glucose; LACT – lactate

### Lipid profile

Lipid profiles improved in response to chokeberry supplementation. In group A2, significant reductions in CHOL (P-value = 0.011), LDL (P-value = 0.005), and TRIG (P-value < 0.001) were observed, along with a significant elevation in HDL (P-value < 0.001). In group A1, an increase in HDL (P-value = 0.006) and a decrease in TRIG (P-value = 0.014) were also observed; however, no significant changes were found in CHOL or LDL. Table 2 presents lipid profile data.

**Table 2.** Lipid profile changes in blood serum of lactating goats after supplementation with *Aronia melanocarpa* pomace

Parameter	Pomace mass per kg feed (group)	Mean ± standard deviation		P-value
		Start	Finish	A1, A2, C Start to finish
TRIG mmol/L	15g (A1)	0.3±0.05	0.2±0.04	0.014*
	30g (A2)	0.3±0.03	0.2±0.03	<0.001*
	control (C)	0.3±0.1	0.4±0.06	0.123
CHOL mmol/L	15g (A1)	3.0±0.47	2.7±0.31	0.076
	30g (A2)	3.0±0.22	2.6±0.37	0.011*
	control (C)	3.2±0.4	3.3±0.38	0.467
HDL mmol/L	15g (A1)	2.1±0.17	2.3±0.13	0.006*
	30g (A2)	2.1±0.35	2.6±0.14	<0.001*
	control (C)	2.1±0.1	2.0±0.17	0.313
LDL mmol/L	15g (A1)	0.8±0.06	0.7±0.10	0.011
	30g (A2)	0.8±0.09	0.6±0.12	0.005*
	control (C)	0.8±0.1	0.9±0.15	0.242

* – statistically significant differences at P-value ≤ 0.05

1 TRIG – triglycerides; CHOL – total cholesterol; HDL – high-density lipoprotein; LDL – low-density lipoprotein

### Macro- and microelements

Table 3 shows a significant decrease in sodium levels in all groups: group A1 (P-value < 0.001), group A2 (P-value = 0.001) and the control group (P-value < 0.001). Potassium and chloride levels did not change significantly. In contrast, magnesium levels decreased significantly in all groups (P-value < 0.05). A significant drop in phosphorus levels was observed only in group A1 (P-value = 0.010). Calcium levels remained stable. Iron concentrations significantly increased in group A1 and group A2 (P-value < 0.001).

**Table 3.** Macro- and microelement concentrations in blood serum of lactating goats after supplementation with *Aronia melanocarpa* pomace

Parameter	Pomace mass per kg feed (group)	Mean ± standard deviation		P-value
		Start	Finish	A1, A2, C Start to finish
K mmol/L	15g (A1)	4.3±0.39	4.2±0.34	0.726
	30g (A2)	4.2±0.56	4.2±0.32	0.746
	control (C)	4.1±0.4	4.2±0.45	0.707
Na mmol/L	15g (A1)	148.5±8.57	134.2±3.16	<0.001*
	30g (A2)	146.3±5.61	137.0±2.71	0.001*
	control (C)	150.3±8.3	137.4±2.57	<0.001*
Cl mmol/L	15g (A1)	110.1±7.24	108.9±2.61	0.614
	30g (A2)	107.6±6.50	108.7±2.78	0.624
	control (C)	110.8±6.1	108.1±1.86	0.265
Mg mmol/L	15g (A1)	1.3±0.15	1.1±0.09	<0.001*
	30g (A2)	1.3±0.07	1.2±0.06	0.035*
	control (C)	1.3±0.1	1.2±0.05	0.002*
P mmol/L	15g (A1)	2.8±0.33	2.3±0.30	0.010*
	30g (A2)	2.2±0.19	2.2±0.32	0.786
	control (C)	2.9±0.4	2.8±0.47	0.815
Ca mmol/L	15g (A1)	2.0±0.34	2.0±0.16	0.488
	30g (A2)	2.1±0.15	2.2±0.11	0.696
	control (C)	2.1±0.4	2.0±0.30	0.511
Fe μmol//L	15g (A1)	21.5±1.29	26.9±2.47	<0.001*
	30g (A2)	20.5±1.31	30.9±1.74	<0.001*
	control (C)	20.7±1.4	21.8±2.58	0.210

* – statistically significant differences at P-value ≤ 0.05

### Aminopeptidase activity

As presented in Table 4, AlaAP activity significantly increased only in group A2 (P-value = 0.007). Also changing exclusively in one group, LeuAP activity significantly decreased only in the control group (P-value = 0.033). In contrast, ArgAP activity significantly decreased in group A1 (P-value = 0.017) and group A2 (P-value < 0.001).

**Table 4.** Aminopeptidase enzyme activity in blood serum of lactating goats after supplementation with *Aronia melanocarpa* pomace

Parameter	Pomace mass per kg feed (group)	Mean ± standard deviation		P-value
		Start	Finish	A1, A2, C
				Start to finish
AlaAP nmol/(mg protein·h)	15g (A1)	4.1±0.36	4.2±0.40	0.727
	30g (A2)	4.0±0.44	4.5±0.48	0.007*
	control (C)	4.3±0.4	4.3±0.38	0.809
LeuAP nmol/(mg protein·h)	15g (A1)	3.0±0.45	2.9±0.24	0.541
	30g (A2)	2.9±0.45	2.7±0.41	0.295
	control (C)	3.0±0.3	2.6±0.26	0.033*
ArgAP nmol/(mg protein·h)	15g (A1)	4.0±0.46	3.5±0.43	0.017*
	30g (A2)	4.8±0.37	4.0±0.44	< 0.001*
	control (C)	4.9±0.4	4.7±0.54	0.381

* statistically significant differences at P-value ≤ 0.05

1 AlaAP – alanyl aminopeptidase, LeuAP – leucyl aminopeptidase, ArgAP – arginyl aminopeptidase

### Lysosomal enzymes

After supplementation ACP activity significantly increased in both group A1 and group A2 (P-value < 0.001). The goats’ β-GRD activity significantly decreased only in group A2 (P-value < 0.001). Activities of β-GAL, β-GLU, and HEX significantly increased in both supplemented groups (P-value < 0.001), while α-GLU and MAN activities significantly decreased in group A1 and group A2 (P-value < 0.05). The obtained values for these activities are set down in Table 5.

**Table 5.** Lysosomal enzyme activity in blood serum of lactating goats after supplementation with *Aronia melanocarpa* pomace

Parameter	Pomace mass per kg feed (group)	Mean ± standard deviation		P-value
		Start	Finish	A1, A2, C
				Start to finish
ACP nmol/(mg protein·h)	15g (A1)	10.9±1.40	15.9±2.23	<0.001*
	30g (A2)	10.7±1.02	19.4±1.28	<0.001*
	control (C)	11.0±1.1	11.3±0.93	0.662
β-GRD nmol/(mg protein·h)	15g (A1)	2.3±0.35	2.0±0.30	0.052
	30g (A2)	2.4±0.47	1.7±0.25	<0.001*
	control (C)	2.4±0.2	2.3±0.22	0.468
β-GAL nmol/(mg protein·h)	15g (A1)	3.4±0.29	4.2±0.26	<0.001*
	30g (A2)	3.4±0.50	4.8±0.32	<0.001*
	control (C)	3.5±0.4	3.6±0.34	0.429
β-GLU nmol/(mg protein·h)	15g (A1)	1.9±0.14	4.7±0.62	<0.001*
	30g (A2)	2.1±0.24	5.2±0.64	<0.001*
	control (C)	2.1±0.3	2.1±0.22	0.856
HEX nmol/(mg protein·h)	15g (A1)	2.9±0.42	5.4±0.51	<0.001*
	30g (A2)	2.6±0.42	5.8±0.78	<0.001*
	control (C)	2.8±0.5	2.8±0.35	0.871
α-GLU nmol/(mg protein·h)	15g (A1)	7.5±0.92	5.5±0.58	<0.001*
	30g (A2)	8.0±0.63	5.1±0.49	<0.001*
	control (C)	7.9±0.7	7.4±0.38	0.111
MAN nmol/(mg protein·h)	15g (A1)	7.6±0.97	6.5±0.76	0.004*
	30g (A2)	7.6±0.43	6.6±0.69	0.013*
	control (C)	7.7±0.9	7.3±0.84	0.279

* – statistically significant differences at P-value ≤ 0.05

1 ACP – acid phosphatase; β-GRD – β-glucuronidase; β-GAL – β-galactosidase; β-GLU – β-glucosidase; HEX – N-acetyl-β-hexosaminidase; α-GLU – α-glucosidase; MAN – mannosidase

### Antioxidant parameters

As shown in Table 6, GSH levels increased significantly in both group A1 and group A2 (P-value < 0.001), with a more pronounced effect in group A2. Significantly more POL content was only found in group A2 (P-value = 0.013). Capacity for DPPH radical scavenging significantly increased in group A2 (P-value < 0.001). Vitamin C concentration significantly increased in group A1 (P-value = 0.039).

**Table 6.** Antioxidant parameters in blood serum of lactating goats after supplementation with *Aronia melanocarpa* pomace

Parameter	Pomace mass per kg feed (group)	Mean ± standard deviation		P-value
		Start	Finish	A1, A2, C
				Start to finish
DPPH % inhibition	15g (A1)	60.1±3.47	62.3±1.60	0.054
	30g (A2)	59.8±1.65	68.7±2.67	< 0.001*
	control (C)	60.2±2.4	60.8±1.10	0.630
Vitamin C mg/100 mL	15g (A1)	0.9±0.12	1.1±0.11	0.039*
	30g (A2)	1.0±0.15	1.1±0.08	0.136
	control (C)	0.9±0.2	0.9±0.12	0.637
GSH μmol	15g (A1)	283.8±46.35	391.3±67.51	< 0.001*
	30g (A2)	284.3±19.57	509.2±44.12	< 0.001*
	control (C)	283.5±32.3	279.6±38.10	0.856
POL mg GAE/mL	15g (A1)	1.6±0.30	1.6±0.14	0.844
	30g (A2)	1.6±0.32	1.9±0.23	0.013*
	control (C)	1.6±0.2	1.6±0.27	0.834

* – statistically significant differences at P-value ≤ 0.05

1 DPPH – 2,2-diphenyl-1-picrylhydrazyl radical; GSH – reduced glutathione; POL – total polyphenols; GAE – gallic acid equivalent

### Correlations between parameters

**Fig 1.**
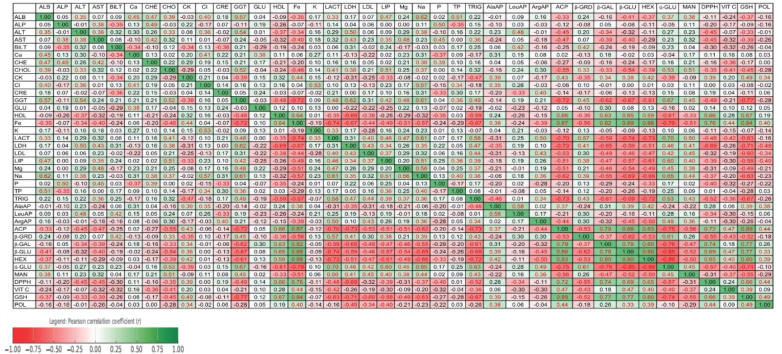
Pearson correlation matrix of selected biochemical, enzymatic, antioxidant and metabolic parameters in high-yielding dairy goats after supplementation with *Arona melanocarpa* pomace

Detailed correlation values are presented in Fig. 1. Positive associations were observed between the hepatic enzymes, ALT correlating with AST (r = 0.38) and GGT (r = 0.54), as well as between lipid and protein parameters such as CHOL correlated positively with ALB (r = 0.39), while ALB strongly correlated with TP (r = 0.51). ALT and GGT were negatively correlated with oxidative stress markers, including GSH (ALT: r = –0.33; GGT: r = –0.77) and DPPH (ALT: r = –0.45; GGT: r = –0.49). ALP was positively associated with AST (r = 0.38), CHE (r = 0.49) and P (r = 0.50), and negatively with BILT (r = –0.35).

Levels of LACT showed positive correlations with α-GLU activity (r = 0.70) and TRIG levels (r = 0.58). The lipid parameters, which were CHOL, LDL and TRIG, showed positive correlations with the hepatic enzymes ALT, GGT and LIP (r = 0.33–0.39) and with carbohydrate metabolism parameters such as α-GLU. Conversely, HDL was positively correlated with GSH (r = 0.67) and DPPH (r = 0.66), and negatively correlated with LDL (r = –0.38), ALT (r = –0.37), GGT (r = –0.48) and TRIG (r = –0.59).

The evaluated LDH activity showed positive correlations with ALT (r = 0.50), AST (r = 0.43), BILT (r = 0.31) and CHOL (r = 0.38), and negative correlations with GSH (r = –0.71), DPPH (r = –0.69), β-GAL (r = –0.65), β-GLU (r = –0.59), and HEX (r = –0.51).

There were noted positive correlations between GSH levels and lysosomal enzymes β-GLU (r = 0.77), β-GAL (r = 0.77) and HEX (r = 0.80), as well as between GSH and HDL (r = 0.67), GSH and ACP (r = 0.89), and GSH and DPPH (r = 0.66). Negative correlations of GSH were observed with LDL (r = –0.60), but negative correlations with LDH (r = –0.71) and Na (r = –0.63). A similar pattern was observed for DPPH, which showed positive correlations with POL (r = 0.44), GSH (r = 0.66) and HDL (r = 0.66), and negative correlations with ALT (r = –0.45), LIP (r = –0.39), LDL (r = –0.32) and TRIG (r = –0.52).

Iron concentration was positively correlated with HDL (r = 0.64), ACP (r = 0.87), GSH (r = 0.84) and DPPH (r = 0.76), and negatively correlated with LDL (r = –0.44), TRIG (r = –0.67) and ALT (r = –0.34). Strong positive correlations were observed among aminopeptidase and lysosomal enzymes, especially between β-GLU and HEX (r = 0.90), and between AlaAP and LeuAP (r = 0.58), AlaAP and ACP (r = 0.37) and AlaAP and GSH (r = 0.39). ArgAP showed strong positive significant correlations β-GLU (r = 0.89), and negative correlations with HEX (r = –0.50) and ACP (r = –0.44).

## Discussion

Dietary supplementation with natural antioxidants, such as the polyphenols found in chokeberries, is receiving increasing attention as a means of improving metabolic health and organ function in farm animals (18, 21). Lactation is a physiologically demanding period associated with enhanced oxidative stress. Effective strategies are being sought to mitigate the effects of this stress, including supplementation with natural antioxidants. Because of its strong antioxidant properties, chokeberry may help reduce oxidative stress and improve liver and kidney function, as well as the regulation of lipid, sugar and protein metabolism. Since research into the effects of chokeberry supplementation in high-yielding dairy goats is still nascent, this study sought to extend knowledge of the potential benefits of this feed additive. Despite their growing importance in milk production, the dairy goat is rarely included in studies of functional nutrition and oxidative stress. Their high metabolic activity and physiological burden during lactation make them an especially valuable model for assessing the effects of antioxidant supplementation. Transcriptional pathways related to antioxidation may be mechanisms of chokeberry bioactive action. The literature indicates that polyphenols, including anthocyanins in chokeberries, can activate nuclear factor erythroid 2-related factor 2 and modulate the expression of inflammatory cytokines such as interleukin 6 (IL-6) and tumour necrosis factor α (TNF-α) (1, 7). These mechanisms may partly explain the observed increases in GSH concentrations and activity of lysosomal and antioxidant enzymes in this study, which are consistent with previous findings on aronia supplementation (14).

Supplementation, particularly in group A2, favourably influenced metabolic and antioxidant parameters, including liver function and repair mechanisms. This suggested a hepatoprotective effect and was further supported by the significant decrease in LDH activity in this group. This reduction may have indicated a protective effect of chokeberry against cellular damage, likely due to reduced oxidative stress. Strong negative correlations with GSH and DPPH confirmed LDH’s relationship with redox balance. Positive correlations of LDH with ALT, AST and BILT suggested a link to liver function, while the correlation with CHOL reflected lipid metabolism. Together, these findings supported the hypothesis that chokeberry reduces cellular damage by improving antioxidant status and stabilising metabolic function. The significant reduction in GGT activity, particularly in group A2, suggested a hepatoprotective and potentially anti-inflammatory effect of chokeberry. Although AST, ALT, and ALP did not change significantly in this group, their overall trends supported this interpretation. These results were consistent with findings from other animal models. Chen *et al*. (6) documented the hepatoprotective effects of chokeberry anthocyanins on the lipid and inflammatory metabolisms in mice, while such effects under oxidative stress were shown in the liver in a cadmium-exposed rat model (14). Renoprotective effects were also reported in a murine ischemia-reperfusion model (11). Chokeberry supplementation may improve lipid metabolism, thermogenesis and gut microbiota composition through the action of polyphenols, as demonstrated in diet-induced obese rats (25). This report would not be inconsistent with improved metabolic efficiency as possibly indicated by the observed reduction in lipase activity in this study.

These findings are also consistent with previous observations from the same animals, in which chokeberry supplementation improved milk quality by increasing antioxidant enzyme activity and polyphenol content, while reducing milk fat levels (21). When considered together with the present results concerning liver, kidney, and oxidative stress markers, this supports the hypothesis of a systemic, multi-organ effect of chokeberry bioactives. In this context, the decline in cholinesterase (CHE) activity observed in the control group may reflect the physiological burden of lactation. However, its stability in the higher-dose group (A2), along with its positive correlations with key metabolic and antioxidant parameters, highlights CHE as a potential marker of systemic homeostasis and further supports the protective effect of chokeberry supplementation.

In the control group, a decrease in ALB, CK and CHE activity was observed, which could be assumed to reflect the physiological burden of lactation and increased oxidative stress. In contrast, the increase in CK activity observed in group A2 may have indicated enhanced muscle metabolism in response to supplementation. The absence of CHE reduction in group A2 further supported the potential protective effect of chokeberry, particularly in maintaining liver function. Albumin, a carrier of thiol groups, neutralises free radicals, and its concentrations correlated with hepatic, lipid and antioxidant parameters in this study, as previously reported in transcriptomic studies on periparturient goats (4, 9). Importantly, the correlation analyses in this study excluded the control group. Physiological changes in this group, associated with lactation, could have introduced artefacts. Excluding the control allowed a more accurate assessment of relationships between parameters in response to chokeberry supplementation.

In the higher-dose group (A2), the lipid profile improved, as indicated by reductions in total cholesterol, LDL, and triglycerides, along with an increase in HDL. These results are consistent with previous findings from experimental animal models, where chokeberry polyphenols have been shown to exert hypolipidemic effects. In rats with diet-induced hyperlipidaemia, administration of chokeberry juice significantly reduced total cholesterol, LDL, and triglycerides (22). Similarly, in streptozotocin-induced diabetic rats, chokeberry supplementation led to a 35–39% decrease in triglyceride levels and normalisation of lipid parameters (19). Correlations of CHOL with liver and antioxidant parameters further support the systemic action of chokeberry. This effect may be partially mediated by modulation of gut microbiota, a system shown to modulate lipid metabolism and oxidative status in response to chokeberry intake (25).

A statistically significant increase in CK activity was observed only in group A2 (P-value < 0.001), whereas a significant decrease occurred in the control group. In group A1, CK levels also declined, although not significantly. These results suggest that the higher dose of chokeberry may enhance metabolic and muscle activity, while the lack of increase in group A1 may indicate a threshold for minimal biological efficacy. Similar findings were reported by Yun *et al*. (23) in young mice supplemented with *Aronia melanocarpa* extract, which enhanced skeletal muscle metabolism and upregulated the expression of myogenic differentiation markers. However, the interpretation of these changes should consider the potential increase in serum creatinine as a possible indicator of renal burden. Supporting this, Li *et al*. (11) demonstrated that anthocyanins extracted from *Aronia melanocarpa* alleviated kidney injury in a murine ischemia–reperfusion model, significantly reducing serum creatinine and the levels of proinflammatory cytokines such as TNF-α and IL-6. The correlations observed between CK and antioxidant parameters in the present study further support the hypothesis of a systemic, multi-organ effect of chokeberry bioactives.

The lack of changes in GLU concentrations suggests that chokeberry did not directly affect carbohydrate metabolism. The observed moderate positive correlation between glucose and calcium may indicate shared regulatory pathways involving insulin secretion, glycolytic enzyme activity, or calcium-dependent signalling influenced by gut microbiota interactions. The literature suggested that chokeberry may have affected host metabolic pathways, including glucose regulation, through gut microbiota-derived short-chain fatty acids (24).

The significant increase in iron concentrations observed in groups A1 and A2 may reflect improved bioavailability or mobilisation in response to chokeberry supplementation. This effect could be linked to the antioxidant and anti-inflammatory properties of chokeberry polyphenols, which have been reported to modulate iron homeostasis indirectly (1, 7). Conversely, the significant reduction in magnesium concentrations across all groups, including the control, likely reflects the physiological burden of lactation and the increased metabolic demands during the peripartum period (4, 9). These mineral fluctuations highlight the complex metabolic changes occurring during lactation and the potential modulating effects of chokeberry supplementation.

The observed positive correlations of LACT with TRIG and α-GLU activity may have indicated concurrent carbohydrate and lipid metabolism associated with anaerobic metabolism. This may have reflected increased enzymatic activity and metabolic adaptation to the energy demands of lactation. The overall decrease in lactate concentrations across all groups, especially in those supplemented with chokeberry, could be interpreted as a sign of improved metabolic balance, greater oxygen efficiency, and reduced metabolic stress. This finding aligned with the concurrent increase in antioxidant parameters such as GSH and DPPH, suggesting a beneficial effect of chokeberry on cellular metabolism and reduced reliance on anaerobic glycolysis. The presence of consolidated positive correlations among lysosomal enzymes possibly manifested a coordinated metabolic response to lactation-induced oxidative stress which was supported by chokeberry supplementation.

The review by González-Gallego *et al*. (8) highlights the immunomodulatory and anti-inflammatory potential of flavonoids, including those derived from fruits such as chokeberry. In this context, changes in aminopeptidase activity (*e.g*. AlaAP, LeuAP and ArgAP) observed following chokeberry supplementation may reflect alterations in protein metabolism and immune function.

Chokeberry significantly affected lysosomal enzyme activity (ACP, β-GAL, β-GLU and HEX), enhancing catabolic and antioxidant potential. While these specific enzyme changes were not examined by Pei *et al*. (16), their findings in a T cell-transfer colitis model supported the anti-inflammatory and antioxidant properties of chokeberry in intestinal inflammation. The suppressive effect on α-glucosidase may be indirectly supported by data from Lim *et al*. (12), who reported a hypoglycaemic effect of chokeberry extract in a high-fat diet model; the effect may be at enzymatic level and be inhibition of carbohydrate digestion and absorption. Although inhibition of α-GLU was not directly assessed, the findings suggest metabolic benefits relevant to carbohydrate digestion and insulin regulation. The inhibitory effect on α-GLU did not result in altered blood GLU levels in the studied animals, possibly because of their physiological metabolic equilibrium. In conditions of impaired GLU homeostasis, such as insulin resistance or high carbohydrate diets, the effect might be more pronounced.

The observed increase in glutathione, polyphenol and vitamin C concentrations, along with enhanced DPPH radical scavenging activity – particularly in group A2 – confirmed the effectiveness of chokeberry in boosting the organism’s antioxidant capacity. The increase in polyphenol concentrations was significant only in group A2 (P-value = 0.013), suggesting a dose-dependent accumulation of phenolic compounds in serum. The absence of changes in group A1 possibly reflected lower bioavailability or higher metabolism at a lower intake. The correlations of these parameters with lysosomal enzyme activity suggested that chokeberry acted both through direct radical scavenging and by supporting enzymatic defence mechanisms. Additional correlations of polyphenols with HDL and ACP, as well as DPPH with CHE and calcium, further supported the systemic nature of the antioxidant effect, linking chokeberry supplementation to improved lipid metabolism, lysosomal function, and mineral balance.

This study assessed a wide panel of physiological, biochemical, and enzymatic parameters to evaluate the effects of chokeberry on key aspects of metabolic function. Although gene expression and cytokine analyses were not included, the results provided a comprehensive overview of the systemic action of chokeberry bioactives. The selection of indicators was tailored to a livestock animal model, considering their relevance in production and nutritional contexts. Future studies should incorporate molecular and proteomic analyses to better understand the mechanisms of chokeberry action, particularly in relation to oxidative, inflammatory and metabolic gene expression. Additionally, the effect of chokeberry on gut microbiota composition and activity should be investigated, as recent reports suggested it may mediate the systemic effects of polyphenols. These findings underscore the need for further research into the molecular mechanisms of chokeberry in the context of intensive animal production systems.

## Conclusion

Dietary supplementation of dairy goats with dried black chokeberry (*Aronia melanocarpa*) pomace, particularly at the higher dose, elicited beneficial effects on metabolic parameters, enzymatic activity and antioxidant capacity. The observed changes indicate a systemic action of chokeberry bioactive compounds, supporting physiological homeostasis during lactation. These findings support the rationale for using chokeberry as a natural feed additive in the nutrition of high-yielding dairy goats, with potential implications for improved animal health and the quality of dairy products.
